# Typicality- and instance-dependent label noise-combating: a novel framework for simulating and combating real-world noisy labels for endoscopic polyp classification

**DOI:** 10.1186/s42492-024-00162-x

**Published:** 2024-05-06

**Authors:** Yun Gao, Junhu Fu, Yuanyuan Wang, Yi Guo

**Affiliations:** 1 School of Information Science and Technology, Fudan University, Shanghai, 200433 China; 2 Key Laboratory of Medical Imaging Computing and Computer Assisted Intervention of Shanghai, Shanghai, 200433 China

**Keywords:** Noisy label, Instance-dependent label noise, Noisy label simulation, Real-world label noise, Polyp classification

## Abstract

Learning with noisy labels aims to train neural networks with noisy labels. Current models handle instance-independent label noise (IIN) well; however, they fall short with real-world noise. In medical image classification, atypical samples frequently receive incorrect labels, rendering instance-dependent label noise (IDN) an accurate representation of real-world scenarios. However, the current IDN approaches fail to consider the typicality of samples, which hampers their ability to address real-world label noise effectively. To alleviate the issues, we introduce typicality- and instance-dependent label noise (TIDN) to simulate real-world noise and establish a TIDN-combating framework to combat label noise. Specifically, we use the sample’s distance to decision boundaries in the feature space to represent typicality. The TIDN is then generated according to typicality. We establish a TIDN-attention module to combat label noise and learn the transition matrix from latent ground truth to the observed noisy labels. A recursive algorithm that enables the network to make correct predictions with corrections from the learned transition matrix is proposed. Our experiments demonstrate that the TIDN simulates real-world noise more closely than the existing IIN and IDN. Furthermore, the TIDN-combating framework demonstrates superior classification performance when training with simulated TIDN and actual real-world noise.

## Introduction

Deep learning neural networks have achieved remarkable performance [1] due to large amounts of labeled data availability. Unfortunately, labeling for medical image classification is often time-consuming and expert-demanding, which could lead to incorrect annotations. Noise labels refer to incorrect annotations, which can originate from inexperienced experts or mistakes made by annotators [2], particularly in endoscopic polyp classification with indistinct features. Noisy labels can mislead deep neural networks due to their strong ability to fit images and labels [3]. Consequently, learning with noisy labels (LNL) methods have been developed. These techniques aim to train neural networks effectively using noisy labels while achieving high accuracy (ACC) on well-annotated test sets. Previous studies [4–8] developed models that handle simulated instance-independent label noise (IIN) [9]. However, their effectiveness is limited in dealing with real-world label noise [10]. Under the IIN paradigm, human-generated noisy labels $\widetilde{Y}$ is only related to the original true labels $Y$, i.e., the noisy transition probability is $P(\widetilde{Y}|Y)$. However, in actual scenarios, label noise is often related to the samples; for example, atypical samples are more likely to be mislabeled. This leads to the concept of instance-dependent label noise (IDN), where the transition probability becomes $P(\widetilde{Y}|Y,X)$, where $X$ denotes the input images. The IDN models the real-world scenario better, resulting in improved handling of real-world label noise compared with the IIN. Therefore, to address the challenge of learning with real-world label noise, it is crucial to simulate and combat it.

**Fig. 1:**
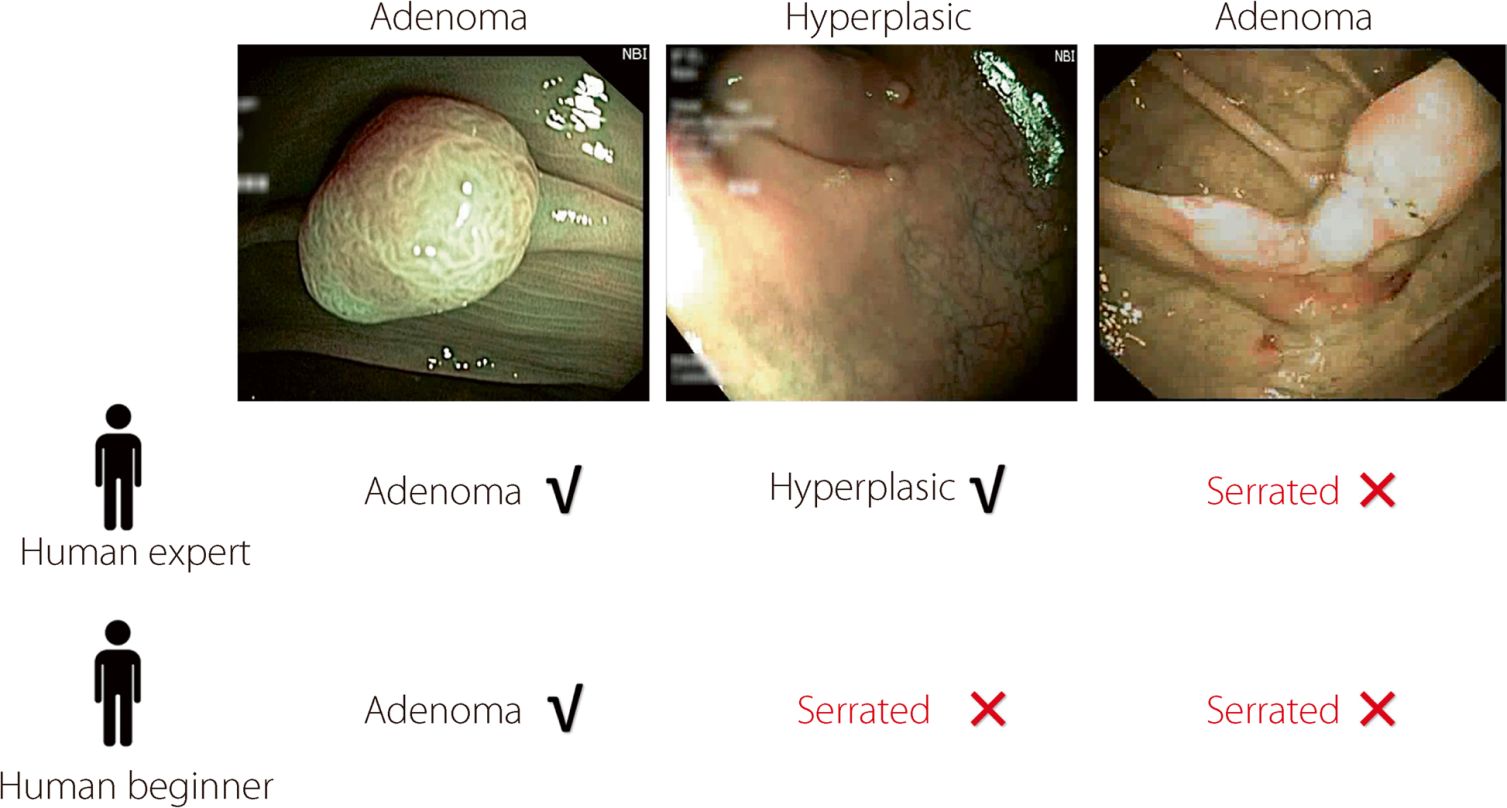
Effect of typicality in real-world label noise. The ground truth above the images is derived from histopathology. The left colonoscopy image displays typical characteristics, whereas the middle one shows atypicality with a small lesion, and the right one is blurred. A human expert provides the first row of real-world noisy annotations, and the second row represents the opinion of a human beginner. Incorrect labels are marked in red color

Methods for simulating label noise can be divided into IIN and IDN. The simulated IIN flips the original labels using a noise transition probability matrix [11–13]. This process depends only on the class of the original label. Classic IIN includes random flipping and pair flipping noise. In the IDN paradigm, the simulated label noise described in ref. [14] converts the pixel value into the probability of flipping labels. This approach combines instances and the probability of flipping; however, it lacks reasonableness and ignores the typicality of the samples. Cheng et al. [15] presented a boundary noise model confined to two-dimensional feature spaces. This approach is overly simplistic for complex, multidimensional spaces and falls short of accurately representing real-world label noise. The current IDN fails to consider the critical factors of typicality, particularly in medical tasks. In practical scenarios, the mislabeling of data often correlates with the typicality of the instance features. Figure 1 demonstrates how beginners might find it challenging to correctly identify small atypical lesions, as shown in the second column. Similarly, the experts and novices may have misclassified a blurred adenoma polyp in the third column.

Methods for combating label noise can be categorized into model-based or model-free approaches based on whether they model the noisy transition distribution from the ground truth to noisy labels. Model-free approaches do not model the noise paradigm (i.e., IIN or IDN). They mainly rely on the “small loss trick” [16], which suggests that the training loss for samples with noisy labels tends to be larger than for those with ground truth. This category includes methods such as MentorNet [17], co-teaching [4], and co-teaching+ [16]. Sample selection methodologies for identifying labels likely to be valid for network training have emerged. Double branch networks [4, 10, 16] have enhanced the selection precision. However, the “small loss trick” is ineffective for the IDN paradigm [14], as neural networks may overfit complex decision boundaries. Semi-supervised learning methods [5, 18, 19] have also been adapted for the LNL problem. These methods leverage the information within the images of noisy samples to assist in selecting and correcting noisy labels. However, these methods do not fully utilize the information in noisy labels, and the correction error for noisy labels remains uncontrolled.

In comparison, model-based methods are deemed more reliable because they theoretically guarantee an optimal classifier for modeling the distribution of true labels. These methods introduce a noisy transition matrix $T\left({\varvec{X}}\right)$, where ${\varvec{X}}$ denotes the raw instances. This matrix represents the transition probability from the latent ground truth to the observed noisy labels. Given oracle ${T}^{*}$, a statistically consistent model can be learned by minimizing the cross-entropy loss reweighted by ${T}^{*}$ [20]. However, the existing model-based studies rely on strong assumptions. Under the IIN assumption, which implies $T\left({\varvec{X}}\right)={T}_{c\times c}$, ref. [6] established a Softmax layer representing the IIN transition channel, which is optimized in an expectation-maximizing manner. Anchor points methods [21], which assume that the most confident samples of neural networks are predicted correctly as anchor points, estimate and fill the simple ${T}_{c\times c}$. Unfortunately, the estimated ${T}_{c\times c}$ of IIN cannot improve real-world noisy labels. Under the complex IDN assumption, Xia et al. [14] assumed that the noisy transition matrix depends only on the parts [22] of the instances rather than the raw images. Part-dependent methods are ineffective for medical images with more complex features and are difficult to compose into parts. Cheng et al. [15] introduced a method designed to be robust to binary boundary noise and validated it in a two-dimensional feature space, which is inapplicable to complex medical image classification tasks. CSIDN [23] estimated $T\left({\varvec{X}}\right)$ according to the confidence of each sample but did not consider overconfidence from neural networks. In addition to the strong assumptions model-based IDN methods mentioned above, these methods overlook the relevance between typicality and the noisy transition matrix, which aligns with the wild.

We introduce typicality- and IDN (TIDN) to simulate real-world label noise and develop a TIDN-combating framework to combat the label noise. A TIDN is generated by disturbing the original labels according to the typicality of the samples. We propose using the distance between the samples and decision boundaries to represent typicality, calculated using a support vector machine (SVM) [24]. In the TIDN-combating framework, we establish a TIDN-attention module to link features and noisy transition matrix. A recursive algorithm was proposed to enable the framework to learn the noisy transition matrix, following the spirit of the expectation-maximization (EM) algorithm. The classification network correctly predicts with corrections from the learned noisy transition matrix. Moreover, we proposed using an instance-independent noisy transition matrix to initialize the instance-dependent matrix in a recursive algorithm.

Our main contributions are as follows:

- We introduce a TIDN to simulate real-world label noise closely. In the TIDN paradigm, atypical samples are more likely to be mislabeled. We propose using the distance between the samples and decision boundaries to represent typicality, calculated using an SVM.
- We propose the TIDN-combating framework to combat label noise. This method establishes a TIDN-attention module that maps features to a per-sample noisy transition matrix. A recursive algorithm is introduced to enable the framework to learn the transition matrix following the EM algorithm. The network could generate accurate predictions by understanding the transition relationship instead of overfitting noisy labels.
- Experiments were conducted to demonstrate that the TIDN closely mirrors real-world label noise compared with existing simulation paradigms. The TIDN-combating framework exhibits superior performance for both simulated and real-world label noise. This is evidenced by the higher test ACC when training with simulated and real-world label noises.

The remainder of this paper is organized as follows. Methods and experimental setups are described in detail in the Methods section. The experimental results are reported in Results section to demonstrate the effectiveness of the proposed method. In the Discussion section, we provide an extended discussion.

## Methods

**Fig. 2:**
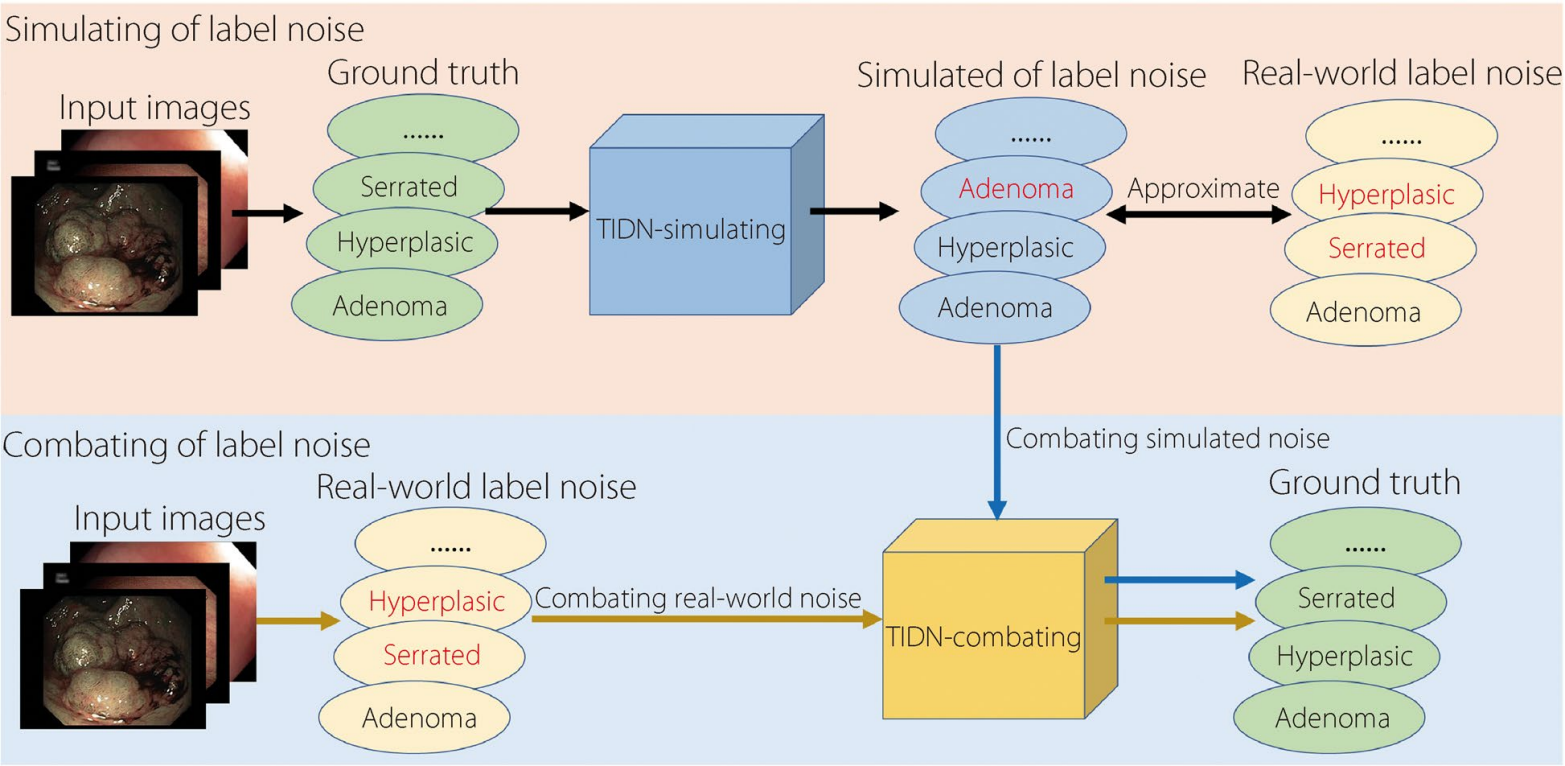
Workflow of the proposed methods. The ellipses represent labels, and the red labels denote the incorrect ones

The workflow of the proposed methods is depicted in Fig. 2. To address the problem of combating real-world label noise, we first seek a simulated label noise to approximate the real world. After that, we design a TIDN-combating framework to combat the well-simulated label noise. With the success in combating well-simulated noise, this framework can also address real-world label noise.

### Preliminaries

In a $C$-class classification task, we are provided with $N$ training pairs ${\left\{\left({x}_{n}, {\widetilde{y}}_{n}\right)\right\}}_{n=1}^{N}$ and $M$ testing pairs ${\left\{\left({x}_{n},{y}_{n}\right)\right\}}_{n=1}^{M}$, where ${x}_{n}$ represents the input medical images and ${\widetilde{y}}_{n}{,y}_{n}\in \left\{1,\dots ,C\right\}$ are the corresponding real-world noisy labels and ground truth, respectively.

The simulation objective is to generate instance-dependent noisy labels ${{y}^{\mathrm{^{\prime}}}}_{n}$ that are closely aligned with the real-world noise ${\widetilde{y}}_{n}$. Under the IIN paradigm, ${{y}^{\mathrm{^{\prime}}}}_{n}$ depends solely on the original true label, ${y}_{n}$. The probability that the generated noisy label belongs to a certain class *j* is $P({{y}^{\mathrm{^{\prime}}}}_{n}=j|{y}_{n}=i)$. Under the IDN paradigm, ${{y}^{\mathrm{^{\prime}}}}_{n}$ depends on ${y}_{n}$ and the input image ${x}_{n}$. The corresponding probability of flipping is $P\left({{y}^{\mathrm{^{\prime}}}}_{n}=j|{y}_{n}=i,{x}_{n}\right)$.

The objective of combating labels is to train a deep neural network classifier using the pairs ${\left\{\left({x}_{n}, {{y}^{\mathrm{^{\prime}}}}_{n}\right)\right\}}_{n=1}^{N}$ for it to perform well on the test set ${\left\{\left({x}_{n},{y}_{n}\right)\right\}}_{n=1}^{M}$.

### Simulating the TIDN

**Fig. 3:**
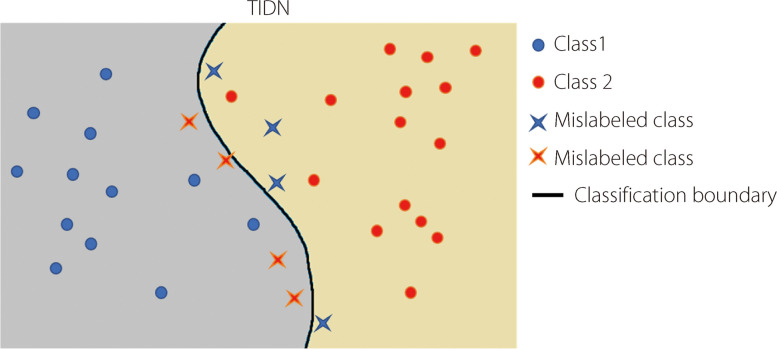
TIDN. In the TIDN paradigm, samples closer to the classification boundary are considered to have weaker typicality, making their labels prone to be mislabeled. In practical scenarios, the feature space extends beyond just two dimensions

Given a dataset ${\left\{\left({x}_{n},{\widetilde{y}}_{n},{y}_{n}\right)\right\}}_{n=1}^{N}$, we generated a simulated ${{y}{\prime}}_{n}$ that could be in close proximity to the real-world noise ${\widetilde{y}}_{n}$ under the IDN paradigm. In actual medical labeling scenarios, instances with typical characteristics are less likely to be mislabeled than those with atypical characteristics. Based on this observation, we propose a method that converts the per-sample distance from the classification boundary into the probability of label disturbance. Figure 3 presents a simplified illustration of the proposed TIDN model. This highlights that samples located at the classification boundaries are susceptible to mislabeling. However, it is important to note that the feature space often has a higher dimensionality in image classification tasks.

An SVM was used to calculate the classification boundary within the feature space explicitly. The boundary hyperplanes, as defined by the “one versus rest” SVM approach [24], are denoted as ${H}_{i}$, where $i\in \{1,..,C\}$ represents the classes. The Euclidean distance to ${H}_{i}$ of each instance is denoted as ${d}_{ti}$*,* where $t\in \{1,..,N\}$ denotes the instances*.* The probability of an instance label being disturbed was then established using the following equation:

1 $$\begin{array}{c}{P}_{t}=1-{e}^{-\lambda {|d}_{tj}|}\end{array}$$

where $j=\underset{i\in\{1,..,C\}}{argmax}\;d_{ti}$. The maximum distance from the *C* channels is translated into the probability of label flipping for the *t*-th sample. Equation (1) ensures that the greater the distance of sample *t* from the hyperplane, the higher the likelihood of label flipping owing to its lower typicality. $\lambda$ is a hyperparameter for controlling the noise ratio of the simulated noisy dataset. After identifying the sample labels flipped using Eq. (1), we determine the specific class to which these labels are flipped. This process involves

2 $$\begin{array}{c}P\left({y}^{\mathrm{^{\prime}}}=j|y=i\right)=Softmax\left({\{d}_{ti}|i\ne j\}\right)\end{array}$$

where *i* represents the class of the original true label *y*, and *j* represents the class of the noisy label ${y}^{\mathrm{^{\prime}}}$ after flipping. $"i\ne j"$ ensures labels do not flip to their original class. The Softmax function can transform a C-dimensional distance into a probability distribution of length C with a sum of one.

### Combating label noise

#### TIDN-combating

Having successfully simulated a TIDN that closely mirrors real-world scenarios, we introduce the TIDN-combating framework. Let ${\varvec{X}}\in {\mathbb{R}}^{h\times w}$ denotes the input image; $Y,\widetilde{Y}\in {\left\{\mathrm{0,1}\right\}}^{C}$ represent the one-hot latent ground truth and observed labels, respectively. Let ${\ell}$ represents the cross-entropy loss for classification, and let $\theta$ denotes the parameters of the classification network. Directly minimizing ${\mathbb{E}}_{{\varvec{X}},\widetilde{Y}}\left[{\ell}\left({f}_{\theta }\left({\varvec{X}}\right),\widetilde{Y}\right)\right]$ leads deep networks to memorize the noisy label. To learn the correct distribution guided by ground truth $Y$, the oracle noisy transition matrix ${T}^{*}\left({\varvec{X}}\right)=$ $P\left(\widetilde{Y}|Y,{\varvec{X}}\right)$ is introduced, as minimizing ${\mathbb{E}}_{{\varvec{X}},\widetilde{Y}}\left[{\ell}\left({T}^{\boldsymbol{*}}{f}_{\theta }\left({\varvec{X}}\right),\widetilde{Y}\right)\right]$ leads to the same effect of minimizing ${\mathbb{E}}_{{\varvec{X}},Y}\left[{\ell}\left({f}_{\theta }\left({\varvec{X}}\right),Y\right)\right]$. Here, we introduce the structure of the TIDN-combating framework and its corresponding recursive algorithm, illustrating the construction of ${T}^{*}\left({\varvec{X}}\right)$. With the modeling of ${T}^{*}\left({\varvec{X}}\right)$, the fitting of the observed $\widetilde{Y}$ leads to the fitting of the latent $Y$.

**Fig. 4:**
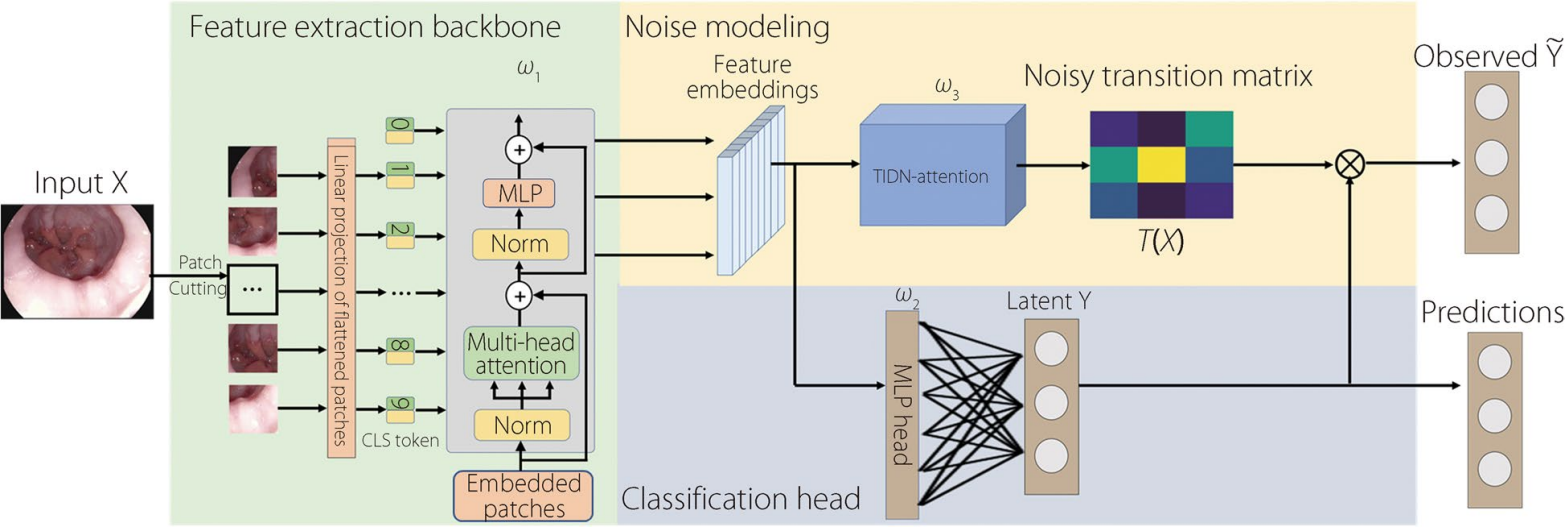
Overview of the TIDN-combating framework. ${\omega }_{1}$: The vision transformer (ViT) [25] backbone follows the original setup with residual connections. ${\omega }_{2}$: The fully connected head of ViT. ${\omega }_{3}$: Parameters of a TIDN-attention block. $T({\varvec{X}})$: Per-sample noisy transition matrix with a $N\times Class\times Class$ dimension. The observed noisy label ${\widetilde{Y}}_{N\times 1}$ is from multiplying ${T({\varvec{X}})}_{N\times C\times C}$ and ${Y}_{N\times 1}$. At the testing phase, the noise modeling phase is removed; the feature extraction backbone and classified head output the final prediction expected to be ground truth

An overview of the TIDN-combating framework is presented in Fig. 4. During the training stage, the feature extraction backbone ${\omega }_{1}$ outputs embedded *F* features. Classification head ${\omega }_{2}$ is expected to predict ground truth $Y$, and the noise modeling phase is expected to construct the mapping from the embedded features to the instance-dependent noisy transition matrix $T({\varvec{X}})$, which is an intermediate product rather than a given parameter [6]. The observed $\widetilde{Y}$ is calculated by multiplying $T({\varvec{X}})$ with $Y$. In the testing phase, the predictions are output through ${\omega }_{1}$ and ${\omega }_{2}$.

#### Structure of the TIDN-attention

**Fig. 5:**
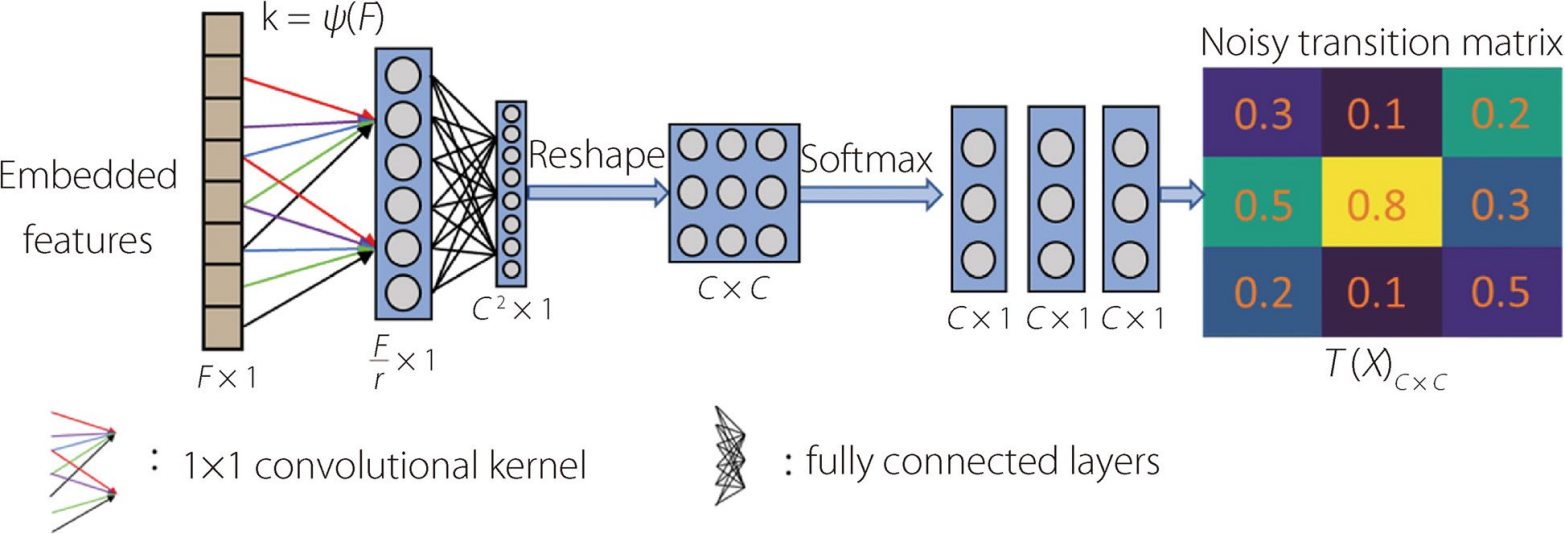
Structure of ${\omega }_{3}$: “TIDN-attention.” The embedded features are first down-sampled by a $1\times 1$ convolutional layer with adaptive kernel size *k*. The number of channels is reduced by $r$. The fully connected layer transfers the results to ${C}^{2}\times 1$ vector, which is subsequently reshaped to a $C\times C$ matrix. Softmax operation is then performed by columns of the matrix to make columns represent $P\left(\left.\widetilde{Y}\right|Y,{\varvec{X}}\right)$. Finally, $C$ of $C\times 1$ column vectors jointly form the typicality- and instance-dependent noisy transition matrix $T({\varvec{X}})$ with a dimension of $C\times C$. The activation function for a $1\times 1$ convolutional and fully connected layers are the rectified linear unit (ReLU) and Sigmoid, respectively

To build the learning pathway from the features to a per-sample noisy transition matrix, the TIDN-attention includes a $1\times 1$ convolutional layer [26] and a fully connected layer, as depicted in Fig. 5. This architecture is aptly termed ‘attention,’ as it extracts a set of optimizable coefficients from the features, which are then applied multiplicatively to $Y$. Notably, $Y$ is also obtained through features using classification head ${\omega }_{2}$.

Specifically, a convolutional layer was used to downsample the features. The kernel size of the $1\times 1$ convolutional layer is set according to $k=\psi \left(F\right)={\left|\frac{{{\text{log}}}_{2}\left(F\right)}{\gamma }+\frac{b}{\gamma }\right|}_{odd}$, where ${\left|t\right|}_{odd}$ indicates the nearest odd number of $t$*.* In this study, we set $\gamma =2,b=1$, in accordance with the default setting outlined in ref. [27] to capture local cross-feature interaction. The downsampled features were then activated by the ReLU function, which is mapped to a ${C}^{2}\times 1$ vector through fully connected layers, where the activating function is a Sigmoid function. ${C}^{2}\times 1$ vector was then reshaped to align with the correct dimension of the noisy transition matrix, and the columns were subjected to a column-wise Softmax operation to align with the definition of the noise transition matrix columns, which represent $P\left(\left.\widetilde{Y}\right|Y,{\varvec{X}}\right)$.

#### Recursive algorithm for noise modeling

With the proposed framework, designing a recursive method to estimate $T({\varvec{X}})$ is feasible, following the spirit of expectation maximization. Instead of the EM algorithm, which cannot be directly used in deep networks, the likelihood of *T* and *Y* is alternately optimized in the proposed algorithm. In the training phase, the log-likelihood is

3 $$\begin{array}{c}L\left({\varvec{\omega}}\right)=\sum\limits_{t}^{N}{\text{log}}P({\widetilde{Y}}_{t}\left|{{\varvec{X}}}_{t};{\omega }_{1},{\omega }_{2}\right)\end{array}$$

When latent variable *Y* is introduced, which represents the latent distribution of ground truth, the new log-likelihood becomes

4 $$\begin{array}{c} L\left({\varvec{\omega}}\right)=\sum\limits_{t}^{N}{\text{log}}(\sum\limits_{i}^{C}P({\widetilde{Y}}_{t},{Y}_{ti}|{{\varvec{X}}}_{t};{\omega }_{1},{\omega}_{2},{\omega}_{3}))\end{array}$$

where *C* is the total class number and ${\omega }_{3}$ represents the TIDN-attention parameters. Based on the training data, we aim to find neural network parameter ${\omega }_{1},{\omega }_{2},{\omega }_{3}$ that maximize the likelihood function. We then introduce ${\omega }^{k-1}$ representing parameters in the last turn to perform an expectation maximization process to optimize recursively ${\omega }^{k}$. According to the EM algorithm, the evidence lower bound of the likelihood function can be derived from Jensen’s Inequality

5 $$\begin{array}{c}L\left({{\varvec{\omega}}}^{k}\right)\ge \sum\limits_{t}^{N}\sum\limits_{i}^{C}P\left({Y}_{ti}|{{\widetilde{Y}}_{t},{\varvec{X}}}_{t};{\omega }_{1}^{k-1},{\omega }_{2}^{k-1},{\omega }_{3}^{k-1}\right)\cdot logP\left({\widetilde{Y}}_{t},{Y}_{ti}|{{\varvec{X}}}_{t};{\omega }_{1}^{k},{\omega }_{2}^{k},{\omega }_{3}^{k}\right)\end{array}$$

We denote ${c}_{ti}^{k-1}$, which is an $N\times C$ matrix, as the posterior distribution of the hidden true label, given the parameters in the last iteration as

6 $$\begin{array}{c}{c}_{ti}^{k-1}=P\left({Y}_{ti}|{{\widetilde{Y}}_{t},{\varvec{X}}}_{t};{\omega }_{1}^{k-1},{\omega }_{2}^{k-1},{\omega }_{3}^{k-1}\right)\end{array}$$

As ${c}_{ti}^{k-1}$ is the posterior distribution of the hidden true label, it can be specifically denoted by the parameters in the final turn

7 $$\begin{array}{c}{c}_{ti}^{k-1}=\frac{{T}^{k-1} {\left({{\varvec{X}}}_{{\varvec{t}}}\right)}_{ji}\cdot {Y}_{i}^{k-1}}{\sum_{i=1}^{C}\left[{T}^{k-1} {\left({{\varvec{X}}}_{{\varvec{t}}}\right)}_{ji}\cdot {Y}_{i}^{k-1}\right]}\end{array}$$

where ${Y}^{k-1}=f({{\varvec{X}}}_{{\varvec{t}}};{\omega }_{1}^{k-1},{\omega }_{2}^{k-1}), { T}^{k-1}\left({{\varvec{X}}}_{{\varvec{t}}}\right)=f({{\varvec{X}}}_{{\varvec{t}}};{\omega }_{1}^{k-1},{\omega }_{3}^{k-1})$; *j* refers to the row number where 1 is located in the one-hot label ${\widetilde{Y}}_{t}$. Note that the calculation of ${c}_{ti}^{k-1}$ generated no gradients in the network. As $T({\varvec{X}})$ is generated by ${\omega }_{3}$ and $Y$ is predicted by ${\omega }_{2}$, the second term in Eq. (5) could be divided into two alternative terms:

8 $$\begin{array}{c} logP\left({\widetilde{Y}}_{t},{Y}_{ti}|{{\varvec{X}}}_{t};{\omega }_{1}^{k},{\omega }_{2}^{k},{\omega }_{3}^{k}\right)=logP\left({\widetilde{Y}}_{t}|{{Y}_{ti},{\varvec{X}}}_{t};{\omega }_{1}^{k},{\omega }_{3}^{k}\right)+logP\left({Y}_{ti}|{{\varvec{X}}}_{t};{\omega }_{1}^{k},{\omega }_{2}^{k}\right)\end{array}$$

The final loss function to be optimized in the neural networks can then be written as the negative of the log-likelihood function

9 $$\begin{array}{c} loss=-\sum\limits_{t}^{N}\sum\limits_{i}^{C}{c}_{ti}^{k-1}\cdot \left[logP\left({\widetilde{Y}}_{t}|{{Y}_{ti},{\varvec{X}}}_{t};{\omega }_{1}^{k},{\omega }_{3}^{k}\right)\text{+}logP\left({Y}_{ti}|{{\varvec{X}}}_{t};{\omega }_{1}^{k},{\omega }_{2}^{k}\right)\right]\\ =-\sum\limits_{t}^{N}\sum\limits_{i}^{C}{c}_{ti}^{k-1}\cdot (log{\left[f({{\varvec{X}}}_{{\varvec{t}}};{\omega }_{1}^{k},{\omega }_{3}^{k})\right]}_{ji}\text{+}log\left[f\left({{\varvec{X}}}_{{\varvec{t}}};{\omega }_{1}^{k},{\omega }_{2}^{k}\right)\right])\end{array}$$

where *j* refers to the row number, and 1 is located on the one-hot label ${\widetilde{Y}}_{t}$.

${c}_{ti}^{k-1}$ is obtained using noisy labels and the parameters in the last turn; the first term in Eq. (9) is directly calculated from the $f({{\varvec{X}}}_{{\varvec{t}}};{\omega }_{1}^{k},{\omega }_{3}^{k})$*,* which equals the *i*-th column of $T({\varvec{X}})$. The last term in Eq. (9) is the prediction results of $f({{\varvec{X}}}_{{\varvec{t}}};{\omega }_{1}^{k},{\omega }_{2}^{k})$. The first term in Eq. (9) also represents the expectation log-likelihood function: ${\mathbb{E}}_{y}(logP(\widetilde{Y}|y,{\varvec{X}}))$, and ${\omega }_{1}^{k},{\omega }_{3}^{k}$ are optimized through gradient decent fixing ${\omega }_{2}^{k}$. The latter term in Eq. (9) also denotes Kullback-Leibler divergence between the prior and posterior distribution of latent true labels, and it is optimized by fixing ${\omega }_{3}$. The pseudocode is presented in Algorithm 1.

#### Initialization of parameters

The successful convergence of the network training hinges on a careful and precise initialization of its parameters for both ${\omega }_{2}$ and ${\omega }_{3}$. We initialized $T\left({\varvec{X}}\right)$ with $T$ using the IIN method [7]. Because $T\left({\varvec{X}}\right)$ in our method is an intermediate product of the network and not a directly adjustable parameter, it necessitates the use of a learning approach to initialize $T\left({\varvec{X}}\right)$ using $T$. Under the IIN paradigm, ref. [7] outputs recognized noisy samples using $T$. We utilized the recognized noisy samples to train ${\omega }_{3}$ while fixing ${\omega }_{2}$.

In the proposed framework, $T\left({\varvec{X}}\right)$ is obtained through the propagation paths of ${\omega }_{1}$ and ${\omega }_{3}$, whereas $Y$ is acquired via the pathways of ${\omega }_{1}$ and ${\omega }_{2}$. Therefore, based on the multiplicative relationship $T\left({\varvec{X}}\right)Y=\widetilde{Y}$, the network can learn the noise transition matrix of the IIN method as an initialization by fixing ${\omega }_{2}$ and optimizing ${\omega }_{3}$.

In addition, a warm-up stage is required to learn the initial distribution of $Y$. We set warm-up epochs to optimize $\omega_1$ and ${\omega }_{2}$ while freezing ${\omega }_{3}$, as the samples with noisy labels still benefit neutral networks in an early training stage [28].

#### Final prediction at test phase

Because $T({\varvec{X}})$ could model the transition distribution from the ground truth to the observed noisy labels, the network shown in Fig. 4 fits both the observed noisy label $\widetilde{Y}$ and the latent ground truth. During the training stage, the feature extraction backbone and classification head could be fed with correct supervision; the fitting of $\widetilde{Y}$ leads to the simultaneous fitting of the ground truth. Thus, the noise modeling phase was removed during the test phase, and the remaining feature extraction backbone and classification head output the final classification predictions.

**Figure:**
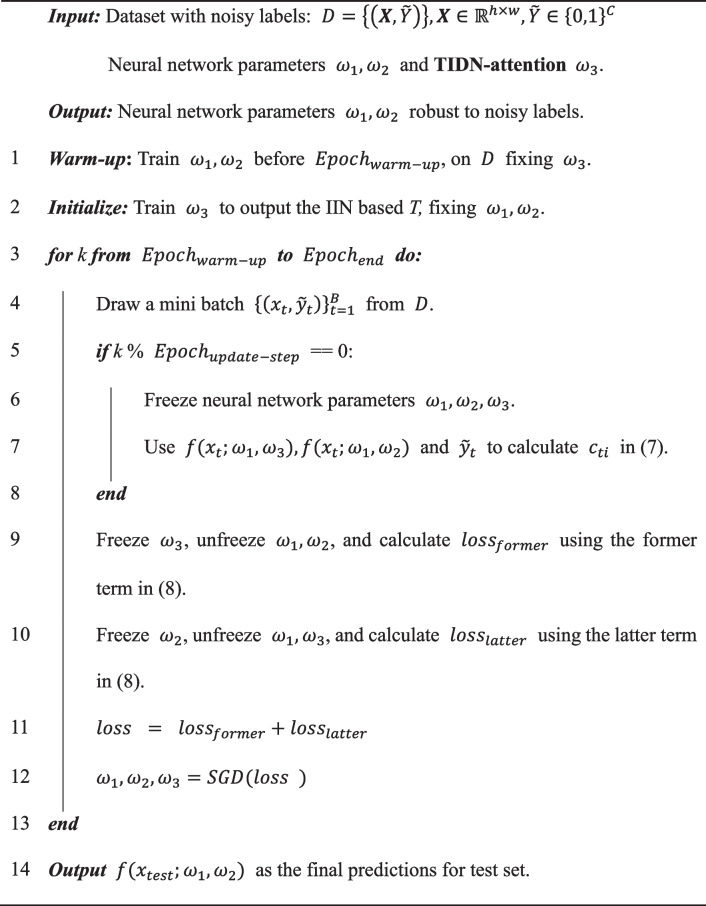
** Algorithm 1** Recursive and alternative optimization for TIDN-attention

### Dataset

We selected two datasets for colonoscopy image polyp classification: Kvasir V2 [29] and a colonoscopy video classification dataset [30]. The public dataset, Kvasir V2, contains 8000 images across eight categories, with 1000 images per category. These categories included dyed resection, esophagitis, ulcerative colitis, and five other classes relevant to polyp characterization. The labels sourced from clinical institutions and experts were considered accurate. The public dataset [30] comprised 152 colonoscopy videos, including 80 adenoma, 30 serrated, and 42 hyperplastic videos, amounting to three lesion types. The video lengths varied from 6 s to 76 s, with an average of approximately 30 s. The labels were derived from the histopathology results and diagnoses by expert doctors or beginners. Histopathology results provided accurate annotations, whereas diagnoses by experts and beginners were considered noisy, with noise ratios of 35.52% and 50.00%, respectively.

We employed a video classification dataset [30] with real-world label noise to validate the proposed method for simulating label noise. In this dataset, the histopathology results were considered the ground truth. The annotations made by the experts and beginners were treated as label noise with noise ratios of 50.00% and 35.52%, respectively. The effectiveness of the noise simulation methods was validated by comparing the similarity between the simulated noise and actual real-world noise.

To verify the ability of the model to combat label noise, we trained it on both simulated and real label noise data. The datasets were divided into training, validation, and test sets at an 8:1:1 ratio. The training set labels were noisy, whereas the validation and test sets contained accurate labels.

### Baselines and metrics

The IIN and IDN were compared with the proposed TIDN. The IIN contains symmetric and pair-flip-label noise [11–13]. For symmetric label noise, the labels of randomly selected instances were uniformly flipped to other classes. For symmetric noise, labels were flipped to neighboring classes for pair-flip noise. For the simulated IDN proposed in ref. [14], the probability of flipping is related to the pixels of the images, thereby generating IDN.

The comparison methods for combating label noise include the IIN and IDN methods. For the IIN methods, co-teaching+ [16] for methods of selecting clean samples, DivideMix [5] for semi-supervised learning, and noise layer [6] for IIN layers, which are similar to our work, were selected for comparison. The part-decomposing method, part-depend [14], and confident-score-based method, CSIDN [15], were selected for the IDN method. The baseline was set as a ViT trained directly on the noisy labels.

The mean total distance is a metric [31] used to measure the difference between the distributions of a real-world and a simulated noisy dataset. Let ${D}_{1}={\left\{{x}_{i},{y}_{i}^{1}\right\}}_{i}^{N}$ and ${D}_{2}={\left\{{x}_{i},{y}_{i}^{2}\right\}}_{i}^{N}$ be the same dataset with two types of noisy labels. The mean total distance between datasets ${D}_{1}$ and ${D}_{2}$ is defined as

$${d}_{Tv}\left({D}_{1},{D}_{2}\right)=\frac{1}{2N} \sum\nolimits_{i=1}^{N}{\Vert {y}_{i}^{1}-{y}_{i}^{2}\Vert }_{1}$$

where ${y}_{i}^{1}$ and ${y}_{i}^{2}$ are soft labels representing probability distributions over $\left\{1,...,C\right\}$.

Test ACC was chosen as the metric for combating label noise. The annotations in the test and validation sets are the ground truth to prove the robustness of LNL. The test and validation sets were blinded during training.

### Implementation details

The ViT [25] was chosen as the feature extraction backbone of our methods for the image classification task, and the video transformer network [32] was chosen as the backbone for the video classification task. During training, the resolution of all input images was adjusted to 224 $\times$ 224, and the pixel values were normalized channel-wise. The dimensions of the embedded features were $B\times 768$, where $B$ is the batch size. Data augmentation was performed by random cropping and vertical flipping.

The network was based on the PyTorch (version 1.9.1) framework and trained on two 12 GB NVIDIA TITAN Xp GPUs. The ViT was optimized using the stochastic gradient descent (SGD) optimizer, whereas the TIDN-attention structure was optimized using the Adam optimizer. The SGD optimizer applied an initial learning rate of 0.003 divided by 0.2 every 10 epochs. The Adam optimizer set a fixed learning rate of 0.003. The image classification task batch size was set to eight, and one for the video classification.

The training set contained noisy labels, and the validation and test sets contained the ground truth. Notably, the output epoch was chosen based on the top training ACC in the last five epochs, and the validation set was blind during training, as we were studying LNL.

## Results

In this section, we describe the experiments conducted on the image classification dataset with simulated label noise and the video classification dataset with real-world label noise. Validation of the simulated TIDN subsection demonstrates that the proposed simulated noise is closer to the real-world noise. Results for combating the TIDN subsection presents the classification performance of the TIDN-attention method in countering simulated noise. Results for combating real-world label noise subsection demonstrates the classification performance of the TIDN-attention method when trained with real-world label noise. Ablation study of TIDN-combating subsection presents an ablation study of the TIDN-attention module and the initialization process.

### Validation of the simulated TIDN

Different approaches for simulating label noise have been applied to colonoscopy video classification datasets containing real-world label noise. The simulated label noise was compared with real-world label noise to evaluate the simulation methods. Table 1 shows the mean total distances between the existing simulated label noise and real-world label noise from a human expert (low noise level with a noise ratio of 35.52%) and a human beginner (high noise level with a noise ratio of 50.00%). The noise ratio of the human annotators was calculated based on the ACC between the ground truth and their annotations. Our simulated TIDN had the lowest mean total distance to real-world noisy labels for both the low noise ratio (0.3440) and high label noise (0.3581) scenarios. Notably, all the simulated label noises align with the noise ratio of the real-world label noise.

**Table 1:** Mean total distances between the simulated and real-world noise labels

Noise type	Expert (35.52%)	Beginner (50.00%)
TIDN (ours)	**0.3440 ± 0.0130**	**0.3581 ± 0.0190**
IDN	0.3797 ± 0.1520	0.3905 ± 0.2210
Pair-flip	0.4197 ± 0.0090	0.4489 ± 0.0080
Symmetric	0.4635 ± 0.0510	0.4343 ± 0.0490

**Fig. 6:**
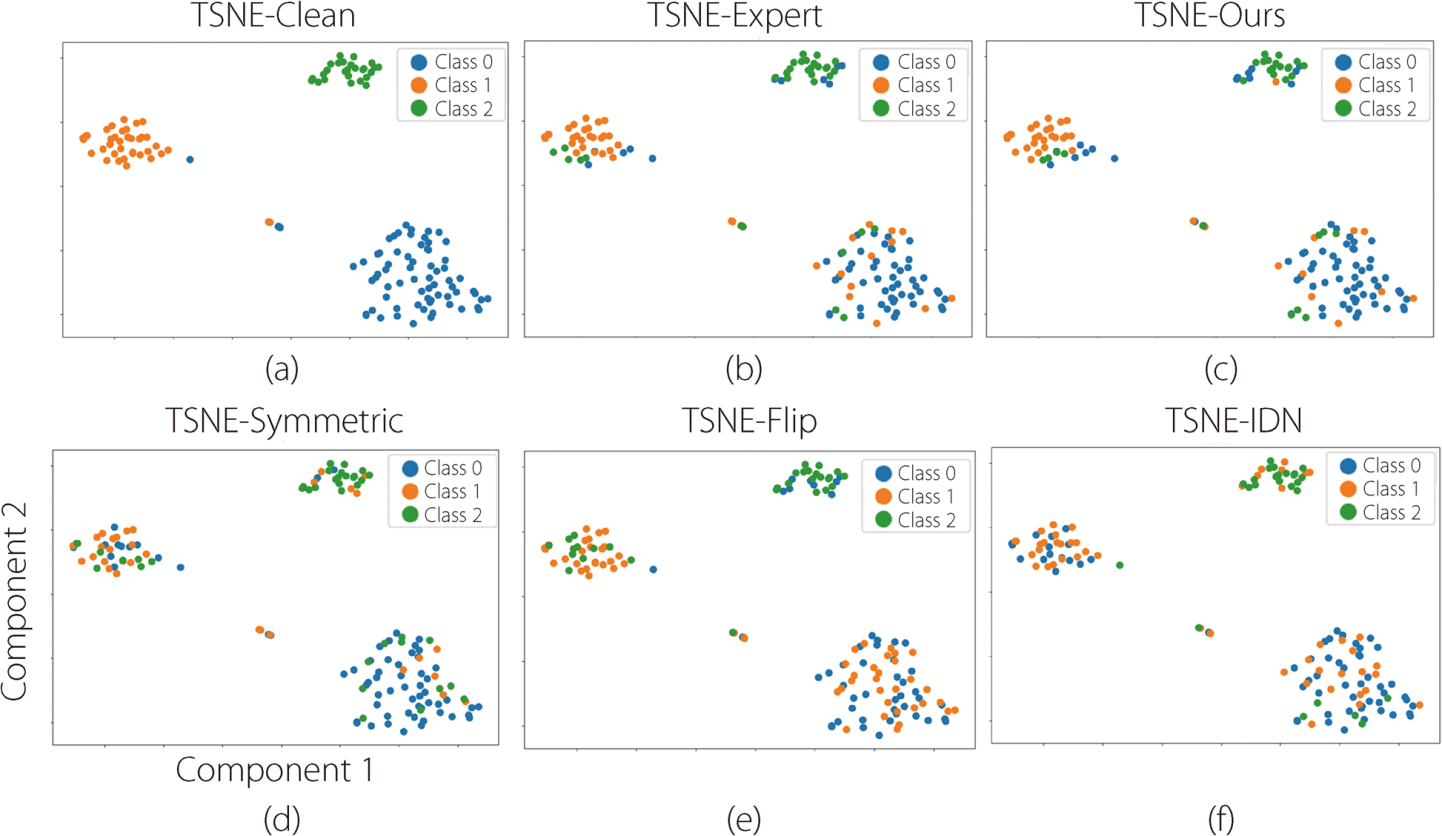
T-SNE map showing the distribution of labels in the feature space for classifying lesions in colonoscopy videos [30]

**Fig. 7:**
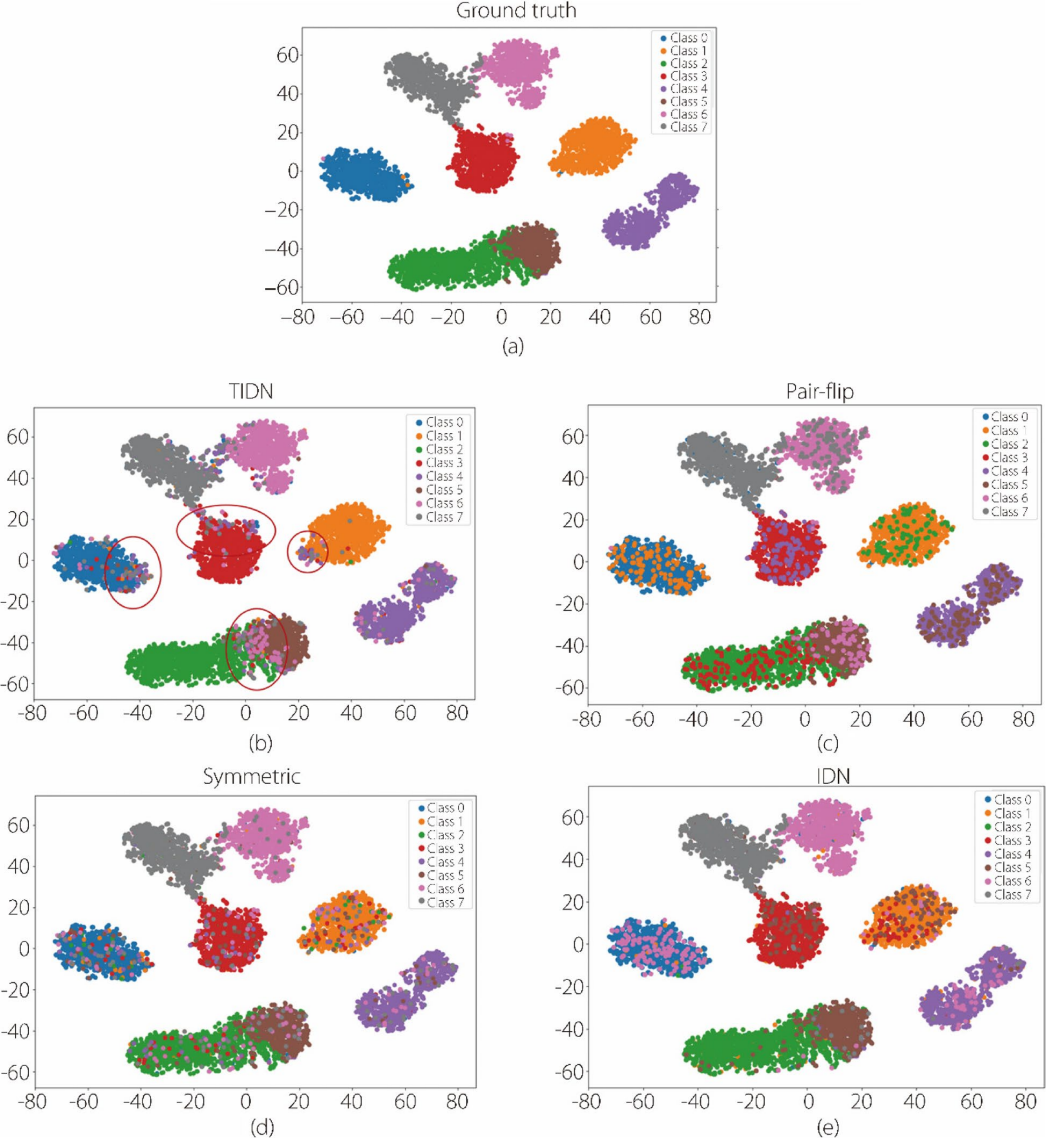
T-SNE map showing the distribution of simulated noisy labels in the feature space for colonoscopy image classification (8 classes). Horizontal and vertical axes represent the two components of the T-SNE plot

The T-SNE map depicting the distribution of instances from different classes is shown in Figs. 6 and 7. In Fig. 6, the two-component T-SNE map shows the distribution of labels in the feature space. The three classes are of three different colors. Different simulated label noises with the same noise ratio (50.00%, aligned with that of a human expert) and ground truth are presented. The human expert label noise was mainly distributed on the edge of the feature map, and the proposed TIDN was the closest to it from the visualization. The simulation results for the colonoscopy classification for the eight classes are presented in Fig. 7, where there is no real-world label noise. The red circle area shows that the disturbed spaces are usually at the edge of the classification boundaries, indicating that atypical samples are more easily disturbed.

### Results for combating the TIDN

Methods for combating label noise were evaluated through test ACC when training with simulated and real-world label noise. The test ACC (top 5) of the different methods used for comparison is summarized in Table 2.

**Table 2:** Test ACC (top 5) on Kvasir V2 dataset labeled with simulated TIDN

Method	Noise ratio = 15% (%)	Noise ratio = 40% (%)	Noise ratio = 70% (%)
Baseline	87.81 ± 1.20	67.82 ± $0$.40	34.82 ± 2.40
Co-teaching+	88.12 ± 0.20↑	72.12 ± 4.20↑	53.13 ± 4.60↑
DivideMix	86.14 ± 1.20↓	70.12 ± 2.10↑	**56.41** ± **3.40**↑
Noise layer	83.12 ± 3.90↓	70.92 ± 3.10↑	36.51 ± 3.10↑
Part-depend	85.21 ± 1.30↓	65.12 ± 2.40↓	35.15 ± 1.40↑
CSIDN	89.14 ± 1.70↑	75.12 ± 5.10↑	46.15 ± 6.90↑
TIDN-attention (ours)	**92.44** ± 1.10↑	**86.23** ± 0.40↑	52.31 ± 2.40↑

Notably, the training set contained only simulated noise, whereas the labels were the ground truths in the test set. The baseline indicates that the ViT is trained directly with the simulated TIDN without any methods to combat label noise. Co-teaching+ , DivideMix, and noise layer ignore the dependence of instances. Part-dependent and CSIDN methods consider the instance dependence of label noise. The TIDN-attention achieves the greatest improvement from 87.81% to 92.44% and 67.82% to 86.23% for the 15% and 40% noise ratios, respectively. Under a 70% noise ratio, DivideMix achieved the highest test ACC of 56.41%, whereas our method achieved 52.31%, compared with the baseline of 34.82%.

**Fig. 8:**
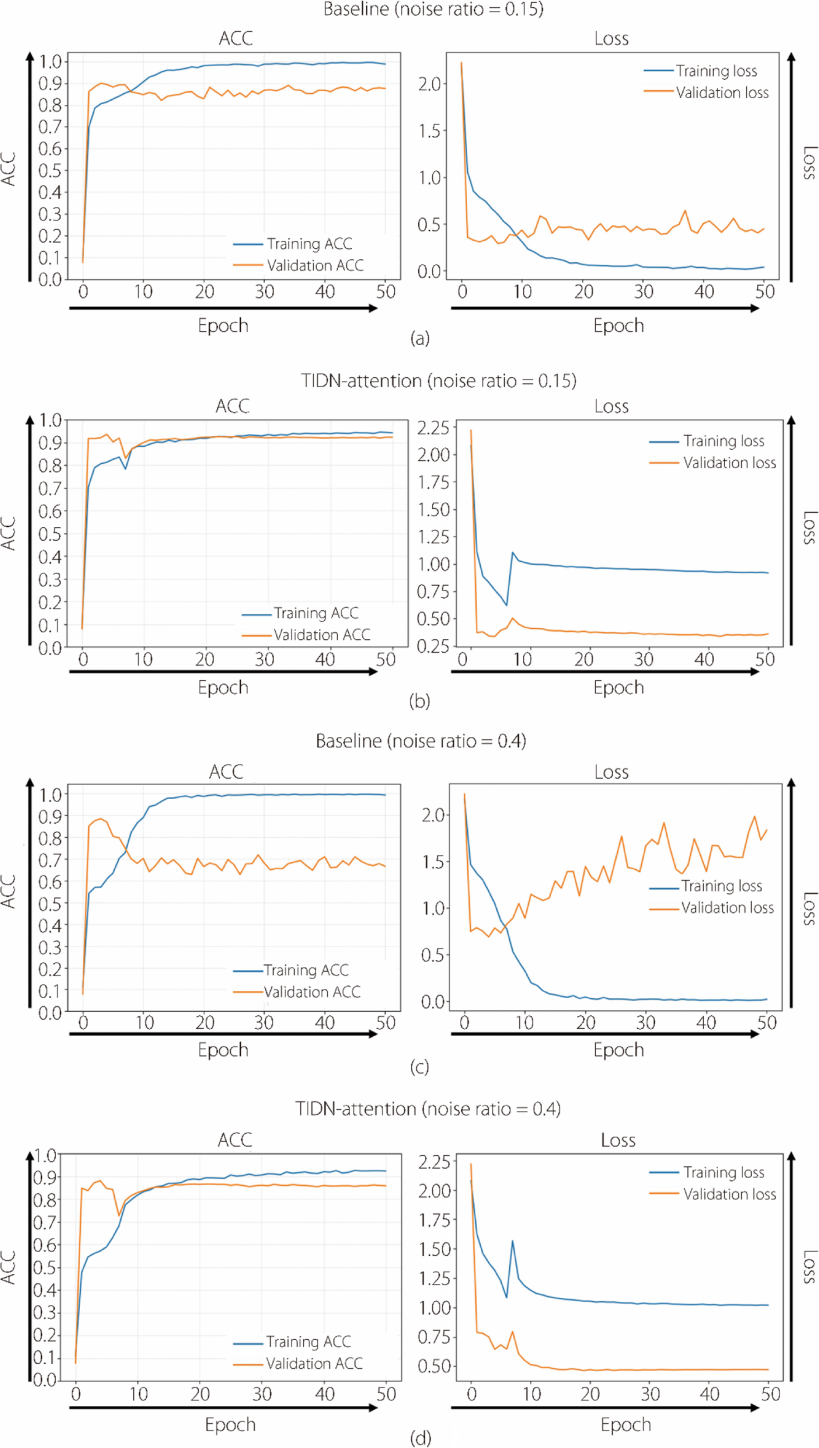
ACC and loss curves during training. The horizontal and vertical axes represent the number of training epochs and the values of ACC or loss, respectively

Figure 8 illustrates the training process of the proposed method, including the curves for ACC and loss during training. The labels in the validation set were accurate, the training set labels were noisy, and the validation set data remained unseen during training. Baseline refers to the classification network being trained directly on noisy data without using methods to counter-label noise. TIDN-attention represents the proposed classification network combating label noise. Figure 8a and c shows that when trained with noisy data, the classification network gradually overfitted noisy labels as the number of epochs increased. This was evidenced by the continuously decreasing loss of the training set, whereas the loss of the validation set initially decreased and then increased. The TIDN-attention method proposed in this study enables the network to fit noisy and accurate labels simultaneously. This is shown in Fig. 8b and d, where the training and validation sets show increased ACC.

### Results for combating real-world label noise

**Fig. 9:**
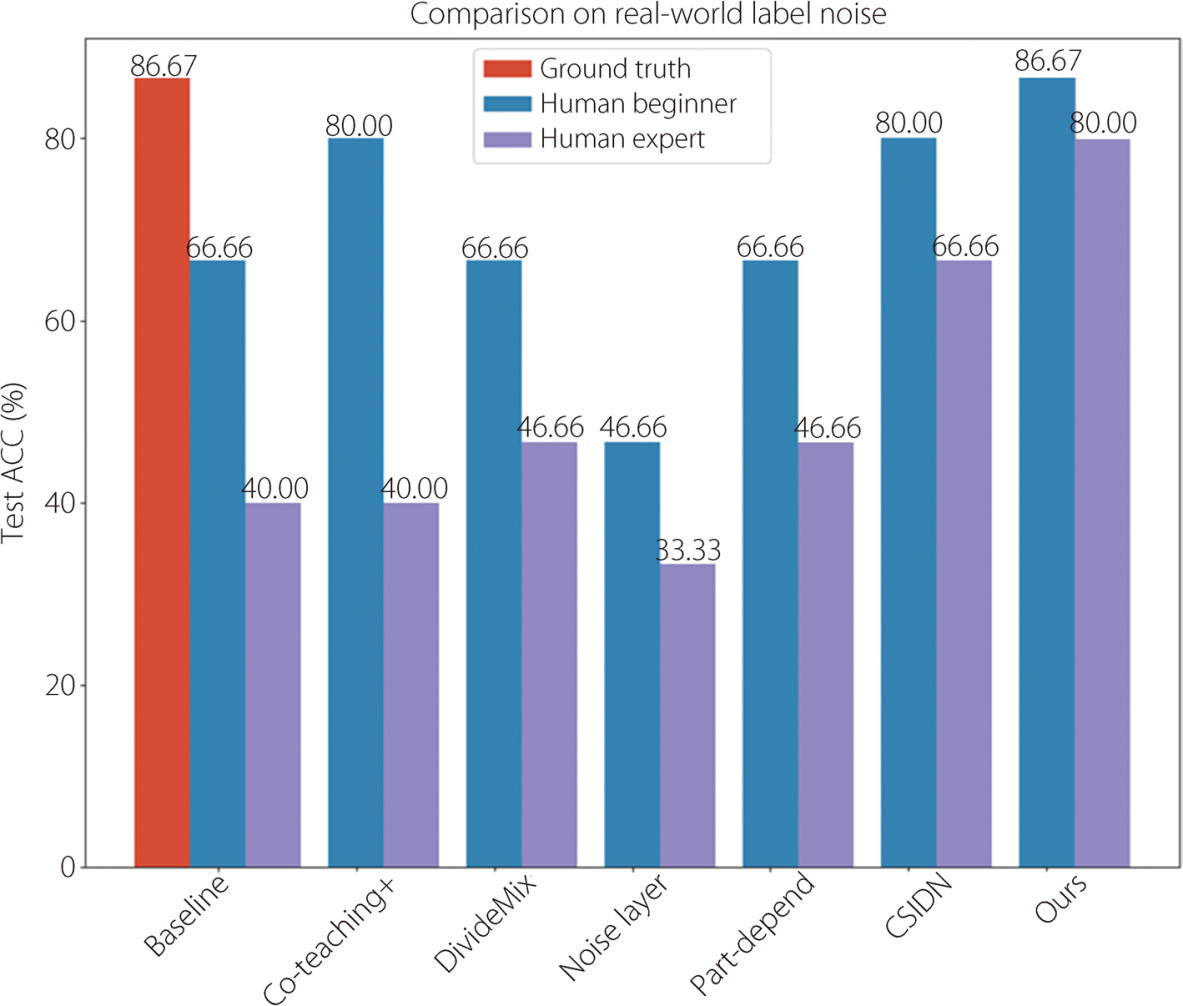
Test ACC of comparative methods under real-world label noise. Baseline refers to a classification network trained directly on noisy labels without anti-noise methods. Ground truth represents the upper bound of LNL, where accurate labels guide the classification network

Results of the real-world label noise are presented in Fig. 9. The test set contained 15 unique videos with ground-truth labels from histopathology. The baseline denotes that the network is trained directly on noisy labels without any methods for combating the label noise. The ground truth is also the upper bound because clean labels guide the network. Our proposed method achieved the same performance of 86.67% as the upper bound when combating real-world label noise based on the opinions of human beginners. It also achieved the highest improvement, from 40.00% to 80.00%, for label noise from human experts. Only the CSIDN designed for IDN effectively improved from 40.00% to 66.66%.

### Ablation study of TIDN-combating

**Fig. 10:**
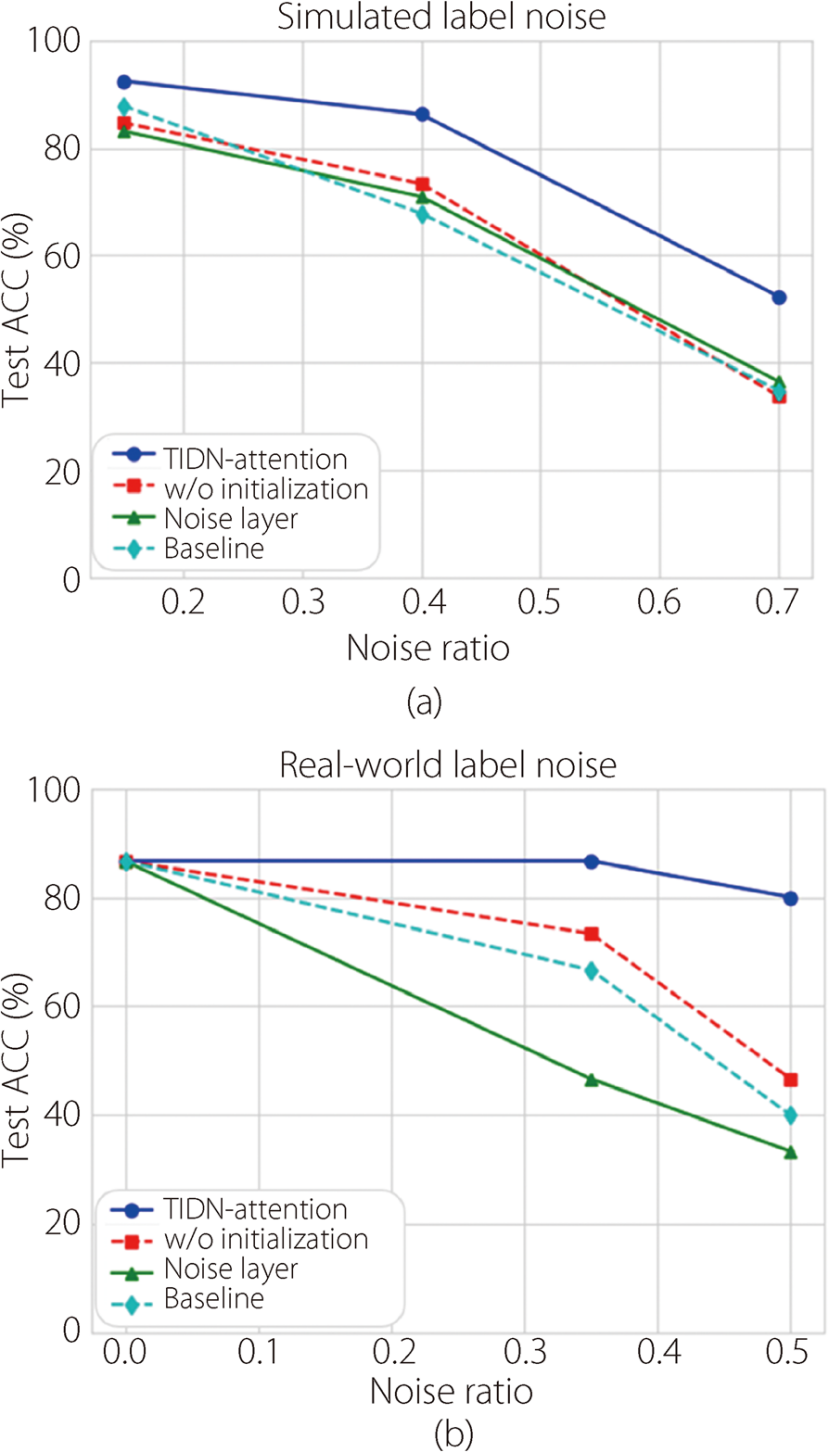
Test ACC in the ablation study across different noise settings

Figure 10 presents the results of the ablation experiments using the TIDN-attention algorithm. The blue solid and red dashed lines represent the results of the proposed TIDN-attention module with and without initialization, respectively. Specifically, without initialization refers to random initialization of ${\omega }_{3}$ and with initialization refers to the method described in Initialization of parameters subsection. The green dashed line represents the scenario in which the noise transition matrix degenerates to IIN [6], assuming *T(****X****)* = *T.* Figure 10a presents the results for simulated noise with noise rates ranging from 15% to 70%, whereas Fig. 10b shows the outcomes for real noise at rates from 35.52% to 50%. Under various noise settings, the proposed method consistently outperformed the ablated methods for the test set ACC.

## Discussion

We introduced a TIDN to simulate real-world label noise and validated this approach by comparing the mean total distance to real-world noise against that of existing simulated noises. Subsequently, we propose the TIDN-combating framework to combat real-world label noise. The performance in combating label noise was validated using simulated and real-world noisy datasets.

In this section, we describe TIDN simulations. Figure 6 illustrates that the simulated TIDN closely resembles real-world label noise. In Fig. 7c, the area marked by the red circle indicates that the samples near the decision boundaries were prone to disturbances. As the T-SNE map represents an abstract feature space, instances on the classification boundaries were effectively identified as atypical. The mean square distances in Table 1 prove that the proposed label noise is the closest to real-world noise. Because the proposed TIDN closely mimics real-world label noise, it can validate the LNL methods without real-world label noise and ground truth data.

The noise resistance performance of the TIDN-combating was demonstrated for real and simulated noise. Table 2 shows that the proposed method combats the TIDN better than the other methodologies, and Fig. 9 proves that it also effectively combats real-world noise. Co-teaching+ , DivideMix, and noise layer ignore the dependence of instances on label noise. Co-teaching+ is ineffective because the small-loss trick does not apply to IDN. DivideMix has the best performance under 70% simulated label noise; however, it performs poorly in other settings. Noise layer is limited because its theory is based on instance-independence assumption. For methods that consider instance dependence, part-depend does not perform as well, and the part-decomposing method does not apply to complex colonoscopy images for medical use. CSIDN has a basic improvement over the baseline; however, it is still limited as the confidence score easily causes networks to fall into overconfidence.

Figure 8 indicates that our method fits both noisy labels (high training ACC) and the latent ground truth (high validation ACC). For the baseline method, the performance on the validation set first increases and then declines as the training ACC increases to the point of overfitting. In contrast, in the training process of TIDN-attention, the validation ACC increases even when the training ACC increases to above 90%. The loss curve shows convergence after a sudden rise in the warm-up and initialization epochs. The training ACC and loss were calculated using noisy labels, whereas the validation ACC and loss were calculated using the ground truth. Training and validation ACC increase simultaneously because our recursive algorithm optimizes the likelihood of *T(****X****)* and the latent ground truth. The structure fits the observed noisy labels while also fitting the ground truth distribution with the assistance of *T(****X****)*. Note that the validation set contains accurate labels, it remains unseen during training in actual LNL scenarios. Despite this, the experiments demonstrate that the proposed method can learn both the distribution of label noise and true labels simultaneously. Therefore, the convergence of the training loss signifies the achievement of a neural network robust to label noise.

Figure 10 shows the results of the ablation study. Comparisons between the noise layer and TIDN-attention highlight the benefits of modeling instance-dependent *T(****X****)* rather than instance-independent* T*. The baseline approach with no modeling of *T* performed poorly. The initialization of *T(****X****)* is inevitable because it outperforms the random initialization methods. This is because initialization restricts the degrees of freedom of *T(****X****)*, enhancing performance.

The limitation of our work lies in the need for better initialization to limit the degrees of freedom of *T(****X****)* or to theoretically tackle the freedom problems for an instance-dependent noisy transition matrix. In addition, our method can be applied to the latest classification methods, such as those based on diffusion models [33, 34], to mitigate the impact of incorrect labels.

## Conclusions

We introduce a novel simulated TIDN for closely approximating real-world label noise. Because TIDN aligns well with real-world scenarios, effectively combating TIDN leads to a combination of real-world label noise. Therefore, we developed the TIDN-combating framework, which includes the TIDN-attention block and a corresponding recursive algorithm. This framework simultaneously fits the observed noisy labels and latent ground truth by modeling a noisy transition matrix, ultimately leading to accurate classification predictions. Our experiments demonstrate that the TIDN closely mimics real-world noise. Furthermore, the TIDN-combating framework achieves superior ACC on the test set annotated with ground truth, whether trained on datasets with simulated or real-world noisy labels.

## Data Availability

The datasets used during the current study are available from the corresponding author upon reasonable request.

## Abbreviations

LNL: Learning with noisy labels
IIN: Instance-independent label noise
IDN: Instance-dependent label noise
TIDN: Typicality- and instance-dependent label noise
SVM: Support vector machine
EM: Expectation-maximization
ViT: Vision transformer
ReLU: Rectified linear unit
SGD: Stochastic gradient descent
ACC: Accuracy
